# Microarray Analysis of Differentially-Expressed Genes Encoding CYP450 and Phase II Drug Metabolizing Enzymes in Psoriasis and Melanoma

**DOI:** 10.3390/pharmaceutics8010004

**Published:** 2016-02-17

**Authors:** Venil N. Sumantran, Pratik Mishra, Rakesh Bera, Natarajan Sudhakar

**Affiliations:** Department of Biotechnology, Dr. M.G.R. Educational and Research Institute University, Chennai, Tamil Nadu 600095, India; patrick9990@gmail.com (P.M.); hellinone0@gmail.com (R.B.); nsudha79@gmail.com (N.S.)

**Keywords:** psoriasis, melanoma, microarray, CYP450, drug metabolism, personalized medicine

## Abstract

Cytochrome P450 drug metabolizing enzymes are implicated in personalized medicine for two main reasons. First, inter-individual variability in CYP3A4 expression is a confounding factor during cancer treatment. Second, inhibition or induction of CYP3A4 can trigger adverse drug–drug interactions. However, inflammation can downregulate CYP3A4 and other drug metabolizing enzymes and lead to altered metabolism of drugs and essential vitamins and lipids. Little is known about effects of inflammation on expression of CYP450 genes controlling drug metabolism in the skin. Therefore, we analyzed seven published microarray datasets, and identified differentially-expressed genes in two inflammatory skin diseases (melanoma and psoriasis). We observed opposite patterns of expression of genes regulating metabolism of specific vitamins and lipids in psoriasis and melanoma samples. Thus, genes controlling the turnover of vitamin D (CYP27B1, CYP24A1), vitamin A (ALDH1A3, AKR1B10), and cholesterol (CYP7B1), were up-regulated in psoriasis, whereas melanomas showed downregulation of genes regulating turnover of vitamin A (AKR1C3), and cholesterol (CYP39A1). Genes controlling abnormal keratinocyte differentiation and epidermal barrier function (CYP4F22, SULT2B1) were up-regulated in psoriasis. The up-regulated CYP24A1, CYP4F22, SULT2B1, and CYP7B1 genes are potential drug targets in psoriatic skin. Both disease samples showed diminished drug metabolizing capacity due to downregulation of the CYP1B1 and CYP3A5 genes. However, melanomas showed greater loss of drug metabolizing capacity due to downregulation of the CYP3A4 gene.

## 1. Introduction

Cytochrome P450 (CYP450) enzymes are a family of microsomal membrane-bound mono-oxygenases which synthesize important endogenous macromolecules and metabolize drugs [1].

About half of the known 57 CYP450 gene families metabolize drugs and xenobiotics, and are targets for drug development. Notably, the remaining 50% of CYP450 genes play vital roles in metabolizing and controlling steady state concentrations of essential lipids and vitamins [1,2]. Indeed, most CYP450-mediated diseases are caused by defects in metabolism of steroids, fatty acids, cholesterol, bile acids, and de-regulation of vitamin D, retinoid, and eicosanoid metabolism [1]. There are several reports on hepatic CYP450 genes, but few studies on their counterparts in the skin. Indeed, the skin is a major site of extra-hepatic CYP450 activity, and several xenobiotics undergo CYP450-mediated degradation, whereas topically-applied drugs undergo CYP450-mediated activation. These CYP450-mediated reactions can result in adverse effects, such as skin sensitization or carcinogenesis [2]. Inflammation is known to suppress expression of specific hepatic CYP450 genes [3]. However, little is known about the effects of inflammation on the expression of genes encoding CYP450 and phase II drug metabolizing enzymes (DMEs) in the normal and diseased skin. Therefore, our aim was to examine expression of genes encoding DMEs during the progression of inflammatory skin diseases, such as psoriasis and melanoma. Accordingly, we analyzed differentially-expressed genes (DEGs) in these diseases using microarray datasets in the Gene Expression Omnibus (GEO) database. We re-analyzed raw data of selected GEO datasets in order to obtain new insights on regulation of genes encoding CYP450 and phase II drug metabolizing enzymes (DMEs) in psoriasis and melanoma relative to normal skin. We undertook this “data re-mining” study because an important paper showed that reuse of public data is a powerful approach to address new questions/hypotheses. This paper also found that approximately 25% of studies used public data to address a biological problem without generating new samples [4].

Psoriasis is characterized by hyper-proliferation of keratinocytes, impaired differentiation, and infiltration of immune cells into the epidermis. Melanomas are aggressive and lethal human cancers which develop from pre-malignant lesions (benign nevi) via oncogenic transformation and proliferation of melanocytes. Our results show that genes controlling levels of vitamin A, vitamin D, cholesterol catabolism, and antioxidant potential were up-regulated in psoriasis versus normal skin. In contrast, genes regulating these processes were down-regulated in melanomas. Interestingly, key genes regulating drug metabolism were down-regulated in both diseases. We explain the impact of these DEGs in the context of differentiation, inflammation, and drug metabolism in the two diseases. The implications of these results for personalized medicine for these diseases are also explained.

## 2. Experimental Section

### 2.1. Methods

GEO is a public, functional genomics data repository which supports MIAME-compliant microarray data. Each GEO dataset represents a curated collection of biologically- and statistically-comparable samples. We analyzed psoriasis and normal skin samples from four GEO datasets (GSE 30999, GSE 14905, GSE41662, and GSE 34248) [5,6,7]. Three other GEO datasets (GDS 1375, GSE 15605, and GSE 46517) with samples from primary melanomas and normal skin were also analyzed [8,9,10]. Table 1 summarizes salient details of each dataset. BRB-Array Tools is an integrated software package for visualization and statistical analysis of raw microarray data [11]. We used the BRB-Array class comparison tool (version 4.3.2) to identify DEGs between two classes (normal versus diseased) from each of the seven GEO datasets in Table 1. Briefly, class comparison data from each dataset was analyzed with the multivariate permutation test computed with 1000 random permutations and a false discovery rate of 1%. From each data set, we obtained a list of genes showing ≥two-fold change in expression between classes with high statistical significance (*p* =10^−4^–10^−7^). KEGG analysis of these gene lists identified genes encoding DMEs. We present and analyze data on genes encoding DMEs which were differentially expressed in at least three of four data sets for psoriasis, and two of three datasets for melanomas. Gene-E software (Broad Institute) was used to visualize gene intensity profiles and generate heatmaps of DEGs from these datasets.

### 2.2. Validation of Methods

For each psoriasis dataset we compared our list of DEGs with those reported by the original authors, and found a significant match. We focused on 14 genes CYP450 and phase II genes encoding DMEs which were differentially expressed in psoriasis versus normal skin. Notably, we identified 11 of these 14 genes in a report which performed meta-analysis on five microarray studies on psoriasis [12]. For melanomas, we identified 11 CYP450 and phase II genes encoding DMEs which were differentially-expressed relative to normal skin. We could not compare these DEGs with data from the original authors, since their entire gene lists were not provided. However, we did confirm differential expression of established biomarkers of melanoma (*CITED1, SPP1, PHACTR1, MITF, FGFR2, PTPRF*) [10] in all three datasets.

## 3. Results and Discussion

**Figure 1:**
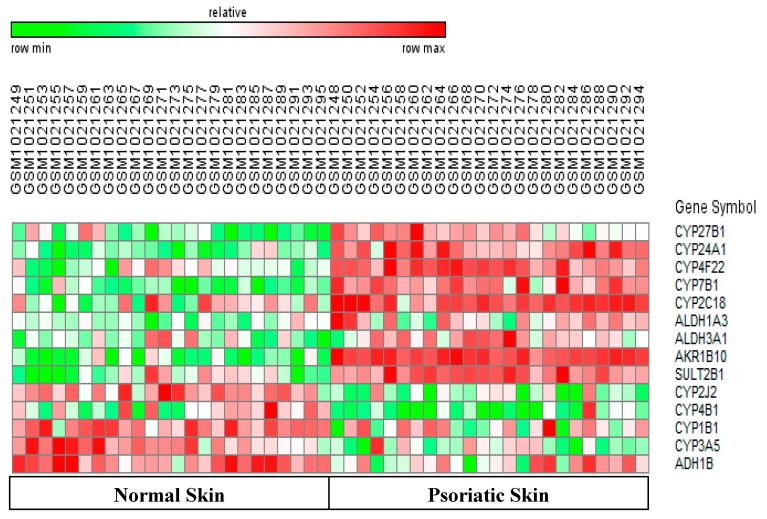
Heatmap of DEGs from GSE 41662.Psoriatic skin showed upregulation of genes controlling metabolism of vitamin D (CYP27B1, CYP24A1), vitamin A (ALDH1A3, AKR1B10), barrier formation (CYP4F22, SULT2B1), antioxidant defense (ALDH3A1, ADH1B), cholesterol catabolism (CYP7B1), and drug metabolism (CYP2C18). Genes metabolizing arachidonic acid (CYP2J2, CYP4B1) and drugs (CYP4B1, CYP1B1, CYP3A5) were down-regulated in psoriasis. Genes with high and low expression are rendered in red and green, respectively.

**Figure 2:**
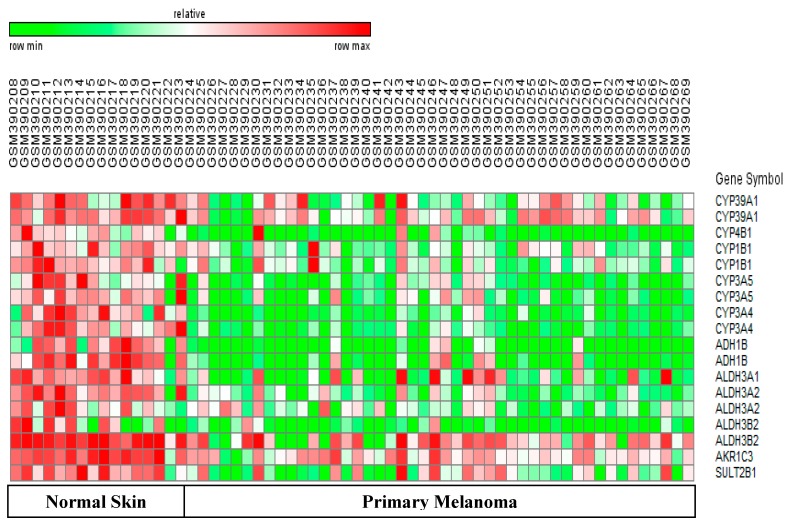
Heatmap of DEGs from GSE 15605. Melanomas showed downregulation of phase II genes controlling vitamin A degradation (AKR1C3), antioxidant defense (ALDH3A1, ALDH3A2, ALDH3B2, ADH1B), and cholesterol catabolism (CYP39A1). Genes metabolizing arachidonic acid (CYP4B1) and drugs (CYP4B1, CYP1B1, CYP3A5, and CYP3A4) were down-regulated in melanomas. Genes with high and low expression are rendered in red and green, respectively. Multiple entries for certain genes arise when different probe sets on the original array recognize an expressed gene.

Nebert *et al.* state that most CYP450-mediated diseases are caused by deregulated metabolism of vitamin D, vitamin A, and defective metabolism of fatty acids and cholesterol [1,2]. This is the first report to show that psoriasis and melanoma samples have opposite expression patterns of key CYP450 and phase II genes when compared with normal skin. We first present data on differentially-expressed CYP450 and phase II genes which regulate metabolism of these vitamins and lipids in each disease. Next, we discuss DEGs regulating the epidermal permeability barrier, antioxidant potential, and inflammation in both diseases. We also analyze the potential impact of genes with opposite patterns of expression in these two diseases. Finally, we discuss genes regulating drug metabolism, and genes which showed similar expression patterns in both diseases. Heatmaps in Figure 1 and Figure 2 show expression of all differentially-expressed genes in psoriasis and melanoma, respectively. Quantitative information for selected genes and the impact of altered expression of these DEGs in psoriasis and melanoma are given in Table 2 and Table 3, respectively.

### 3.1. Increased Turnover of Vitamin D in Psoriasis

Active vitamin D (1,25-dihydroxyvitamin D(3) or calcitriol) regulates calcium homeostasis, suppresses keratinocyte proliferation, and promotes differentiation and growth of the stratum corneum in normal skin*.* Indeed, certain vitamin D analogs are effective drugs for treatment of psoriasis [13]. We observed upregulation of two CYP450 genes regulating vitamin D turnover in psoriasis, Thus, the rate-limiting enzyme for bio-activation of vitamin D (CYP27B1) and an enzyme which degrades vitamin D (CYP24A1), were up-regulated three-fold and eight-fold in psoriasis *versus* normal skin, respectively (Figure 1, Table 2). These data suggest that keratinocytes of psoriatic skin can synthesize active vitamin D and degrade it rapidly. This is consistent with reports of loss of functional vitamin D causing abnormal keratinocyte differentiation in psoriasis [14]. Melanoma samples in this study did not show differential expression of CYP450 genes regulating vitamin D metabolism.

### 3.2. Differential Regulation of Vitamin A metabolism in Psoriasis versus Melanoma

We observed upregulation of two CYP450 genes regulating vitamin D turnover in psoriasis. A similar pattern of gene expression was observed for genes regulating metabolism of all trans-retinoic acid (ATRA) or vitamin A. Thus, psoriasis samples showed a three-fold upregulation of the aldehyde dehydrogenase (ALDH1A3) which synthesizes ATRA, and a 24-fold upregulation of the aldo-keto reductase (AKR1B10), which degrades ATRA [14] (Figure 1 and Table 2). Since the upregulation of AKR1B10 far exceeds that of ALDH1A3, psoriatic skin would have low ATRA levels, which can lead to increased keratinocyte proliferation [15]. Melanomas showed the opposite trend. Thus, Figure 2 and Table 3 show five-fold downregulation of another aldo-keto reductase (AKR1C3) which degrades ATRA. Since active AKR1C3 leads to decreased ATRA synthesis, melanomas can maintain ATRA levels and prevent increased cell proliferation. Interestingly, decreased AKR1C3 expression is reported in certain neural tumors, and melanomas originate from neuro-ectodermal tissue [16].

Overall, ATRA can induce growth arrest and apoptosis in keratinocytes [17]. Therefore, the psoriasis samples could rapidly proliferate due to loss of ATRA caused by upregulation of AKR1B10. In contrast, downregulation of a functionally similar gene in melanomas (AKR1C3), could stabilize ATRA levels and rates of cell proliferation.

### 3.3. Genes Regulating Epidermal Barrier Function Are Up-Regulated in Psoriasis

**CYP4F22:** Most CYP4F enzymes catalyze the ω-hydroxylation of fatty acids such as arachidonic acid, and generate pro-inflammatory compounds. However, little is known about the rare CYP4F22 isoform which showed a significant five-fold upregulation in psoriasis *versus* normal skin. CYP4F22 mutations cause a hyperkeratotic skin disease termed lamellar ichthyosis which is characterized by an abnormal epidermal permeability barrier [18]. Maintenance of the normal barrier requires CYP4F22 mediated conversion of arachidonic acid into specific epoxyalcohols known as hepoxillins. Hepoxillins are precursors of specific ceramides (omega-hydroxyacyl-sphingosine) which covalently bind corneocyte proteins and form the functional permeability barrier in the stratum corneum [19]. Recent studies suggest that upregulation of CYP4F22 can cause aberrant hepoxilin signaling which, in turn, could decrease integrity of the permeability barrier in psoriatic skin [18].

**SULT2B1:** Increased levels of cholesterol sulfate are associated with an epidermal barrier abnormality and a scaling phenotype [20]. Psoriatic skin showed a 3.30-fold upregulation of the sulfotransferase (SUT2B1) which sulfates cholesterol (Figure 1 and Table 2). In contrast, melanomas showed a three-fold downregulation of SULT2B1 (Figure 2 and Table 3). Little is known about SULT2B1 expression in melanomas, but low SULT2B1 expression correlated with decreased proliferation in hepatocellular carcinomas [21].

In summary, there are reports of partial or abnormal differentiation of keratinocytes in psoriasis [13]. Figure 1 and Table 2 also show that psoriatic skin showed upregulation of genes which deplete vitamin D and vitamin A (CYP24A1 and AKR1B10), and lead to abnormal keratinocyte differentiation and epidermal barrier function (CYP4F22, SULT2B1) [18,19,20]. These differentiation-related genes would not be detected in melanomas which contain melanocytes rather than keratinocytes.

### 3.4. Differential Regulation of Antioxidant Potential in Psoriasis versus Melanoma

The ALDH3 family of phase II drug-metabolizing enzymes can oxidize and detoxify lipid peroxide aldehydes produced by oxidative stress. ALDH3A1 (Aldehyde dehydrogenase 3, member A1) is expressed in keratinocytes, and high ALDH3A1 expression and activity were correlated with increased cell proliferation [22]. We observed a 2.0-fold upregulation of ALDH3A1 in psoriasis (Figure 1). The ALDH3A2 and ALDH3B2 isoforms are also involved in lipid detoxification [23]. However, ALDH3A1, ALDH3A2, and ALDH3B2 showed a 2.90-fold, 2.50-fold, and 5-fold downregulation in melanomas, respectively (Figure 2). The ADH1B gene encodes an alcohol dehydrogenase which plays a major role in ethanol catabolism, and oxidizes retinol and lipid peroxides [24]. We observed a 2.80 fold and nine-fold downregulation of the ADH1B gene in psoriasis and melanoma.

In summary, melanomas showed downregulation of three aldehyde dehydrogenases (ALDH3A1, ALDH3A2, and ALDH3B2), which were not down-regulated in psoriasis. Therefore, melanomas would have weaker antioxidant potential than the psoriasis samples in this study.

### 3.5. Differential Regulation of Cholesterol Catabolism in Psoriasis versus Melanoma

**CYP7B1 and CYP39A1:** Our results in Section 3.2 and Section 3.4 showed that genes regulating vitamin A synthesis and antioxidant potential were up-regulated in psoriasis, but down-regulated in melanomas. Interestingly, CYP450 genes controlling cholesterol catabolism also showed opposite patterns of expression in these two diseases. Hydroxysterols are oxidation products of cholesterol esters, and different hydroxysterols with pro-oxidant and pro-inflammatory effects have been identified [25]. For example, the 25-hydroxycholesterol-induced IL-1β secretion in human macrophages, and interfered with the adaptive immune response [26,27]. These findings are significant since psoriasis is an autoimmune disease. The CYP7B1 and CYP39 enzymes convert inflammatory hydroxysterols (24,25, and/or 27-hydroxycholesterol) into less active 7-hydroxysterols, which are then converted into bile salts and excreted [28]. We observed a 2.60-fold upregulation of CYP7B1 in psoriasis versus normal skin (Figure 1 and Table 2). From the viewpoint of personalized medicine, upregulation of CYP7B1 can have beneficial or detrimental effects in psoriasis. Thus, upregulation of CYP7B1 could have an anti-inflammatory effect by converting 25- and 27-hydroxycholesterol into less active forms. However, upregulation of CYP7B1 can be detrimental because it can convert dehydroepiandrosterone (DHEA) into an immunostimulatory metabolite (7α-hydroxy-DHEA) which counteracts the immunosuppressive effects of glucocorticoid therapy [29]. CYP39A1 has similar activity as CYP7B1, but preferentially uses 24-hydroxycholesterol as a substrate. We observed a 3.70-fold downregulation CYP39A1 in melanomas (Figure 2 and Table 3). Little is known about CYP39A1 gene expression in skin, but low CYP39A1 expression correlated with increased inflammation and metastasis in cholangiocarcinoma [30].

Overall, CYP450 mediated cholesterol catabolism may generate anti-inflammatory effects in psoriasis but not in melanomas. This is because increased CYP7B1 expression can inactivate pro-inflammatory 25 and/or 27-hydroxycholesterol in psoriasis. In contrast, downregulation of CYP39A1 can cause accumulation of 24-hydroxycholesterols which could lead to increased inflammation and enhanced melanoma progression.

### 3.6. Regulation of Metabolism of Arachidonic Acid and Xenobiotics in Psoriasis and Melanoma

**CYP2J2:** Certain metabolites of arachidonic acid modulate inflammation. Thus, the CYP epoxygenase (CYP2J2) converts arachidonic acid into epoxyeicosatrienoic acids (EETs) with anti-inflammatory effects in cardiac tissue [31]. We observed a 2.70-fold downregulation of CYP2J2 in psoriasis samples relative to normal skin (Figure 1 and Table 2).If EETs have an anti-inflammatory role in skin, then the downregulation of CYP2J2 can enhance inflammation in psoriasis. Interestingly, a study showed that increased 14,15-EET formation correlated with spontaneous cell cornification [32].Therefore, downregulation of CYP2J2 is consistent with reduced EET production and abnormal cornification of stratum corneum in psoriatic skin [31]. However, the impact of the down-regulated CYP2J2 gene in psoriasis can vary, since the CYP2C8/9 epoxygenases can synthesize EETs and compensate for decreased CYP2J2 expression [31,32].

**CYP4B1:** CYP4 ω-hydroxylases convert arachidonic acid into pro-inflammatory hydroxyl-eicosatetraenoic acids (HETEs). This enzyme also participates in xenobiotic metabolism and causes tissue-specific toxicities in experimental animals [33]. We observed a three-fold and an11-fold downregulation of CYP4B1 expression in psoriasis and melanomas respectively (Table 2 and Table 3). Downregulation of CYP4B1 in both these diseases can potentially decrease inflammation and carcinogenesis. However, the human CYP4B1 isoform is non-functional due to replacement of a proline residue by a serine residue at position 427. This non-functionality was proved by showing that the serine variant of this enzyme could not activate a pro-toxin termed 4-ipomeanol [34]. Therefore, we checked the sequences of the Affymetrix probesets for CYP4B1 in the datasets we studied. We found that these probes recognized the inactive serine variant of human CYP4B1. Therefore, the downregulation of CYP4B1 is unlikely to affect the metabolism of drugs and xenobiotics in the psoriasis and melanoma samples of this study.

### 3.7. Comparative Metabolism of Vitamins and Lipids in Psoriasis and Melanomas

This is the first report to show that psoriasis and melanoma samples have opposite patterns of expression of key CYP450 and phase II DME genes when compared with normal skin. The different expression patterns of these genes may arise because regulation of metabolism of vitamins and lipids are intrinsically different in the cell types affected in psoriasis and melanomas (keratinocytes and melanocytes). Whereas psoriatic skin showed upregulation of genes controlling metabolism of vitamin A and cholesterol catabolism, melanomas showed downregulation of CYP450 and phase II genes regulating these same processes*.* There are five important differences in the patterns of expression of these genes in these two diseases. First, psoriasis samples potentially have lower levels of vitamin A than melanoma samples, due to differential regulation of genes encoding enzymes which degrade vitamin A (AKR1B10 and AKR1C3). Psoriasis samples also showed lower levels of vitamin D due to upregulation of the gene controlling degradation of vitamin D (CYP24A1). Depletion of vitamins A and D in psoriatic skin is consistent with the literature [14]. Second, the inactivation of reactive aldehydes was more efficient in psoriasis due to upregulation of ALDH3A1, whereas melanomas showed weaker antioxidant defense due to downregulation of the ALDH3A1, ALDH3A2 and ALDH3B2 genes. Third, the upregulation of CYP7B1 may enable psoriatic skin to inactivate inflammatory hydroxysterols more efficiently than melanomas, which showed downregulation of the functionally similar CYP39A1 gene. The fourth finding is that psoriasis samples potentially have increased inflammation and impaired cornification of the stratum corneum due to decreased CYP2J2 expresssion and levels of epoxyeicosatrienoic acids (EETs) [31,32]. While genes regulating metabolism of key vitamins and lipids showed opposite expression patterns in these diseases, it is intriguing that two major CYP genes regulating metabolism of drugs and xenobiotics (CYP1B1 and CYP3A5) were downregulated in both diseases. In addition, melanoma samples showed downregulation of the CYP3A4 gene. Therefore, the fifth important finding is that psoriasis samples have greater drug metabolizing ability than melanomas. Before these genes are discussed, we discuss drug metabolizing genes which were differentially expressed in either psoriasis or melanoma.

### 3.8. Differentially-Expressed Drug Metabolizing Genes in Psoriasis

**CYP2C18:** The CYP2C18 gene is a member of the CYP2C family which metabolizes approximately 20% of clinically used drugs [35]. We found a 4-fold upregulation of CYP2C18 in samples of psoriasis relative to normal skin (Figure 1 and Table 2). Little is known about the drugs metabolized by CYP2C18. However, this enzyme can transform retinol into metabolites which inhibited proliferation of several normal and neoplastic cells *in vitro* [36]. Therefore, the upregulation of the CYP2C18 gene may lead to reduced keratinocyte proliferation in psoriasis.

### 3.9. Differentially-Expressed Drug Metabolizing Genes in Melanoma

**CYP3A4:** CYP3A4 is the most abundant hepatic P450 enzyme in humans, and is known to metabolize at least 50% of commonly used drugs. The catalytic activity of CYP3A4 shows large inter-individual variability and can cause significant differences in pharmacokinetic profiles of a given anticancer drug amongst patients [37]. We observed a three-fold downregulation of CYP3A4 in melanomas (Figure 2, Table 3) which, in turn, could lead to a significant decrease in drug metabolizing capacity of melanomas.

### 3.10. Downregulated Drug Metabolizing Genes in Psoriasis and Melanomas

Although psoriasis and melanoma have very different underlying molecular mechanisms and pathologies, we observed downregulation of genes encoding two major drug metabolizing enzymes (CYP1B1 and CYP3A5) in both diseases. Details on each gene are given below.

**CYP1B1:** This gene is overexpressed in several tumors, and CYP1B1-activated pro-drugs and CYP1B1 inhibitors are being developed as anti-cancer therapies [38]. Although CY1B1 is a major CYP gene in human skin, [2,39], little is known about its role in psoriasis and melanoma. Immunohistochemical data confirmed expression of CYP1B1 protein in the epidermis [40], and studies comparing wild type and CYP1B1-null mice proved that carcinogens, such as DMBA (dimethylbenzathracene), induce CYP1B1 expression, skin hyperplasia, and tumors [41]. These abnormalities suggested that increased CYP1B1 expression would play a role in the pathogenesis of psoriasis and melanomas. However, as reported by others [12,42], we observed a 2.70-fold and 4-fold downregulation of CYP1B1 in psoriasis (Figure 1 and Table 2) and melanomas (Figure 2 and Table 2) respectively. The downregulated CYP1B1 in psoriasis and melanoma could occur due to lack of upregulation of known upstream inducers of CYP1B1 (AhR or Aryl hydrocarbon receptor and TNF-α) in these samples [38,39]. Downregulation of CYP1B1 in psoriasis and melanomas can protect skin from activation of many environmental mutagens and also prevent estrogen-dependent hormonal carcinogenesis [38,39]. In addition, CYP1B1 depletion may have an anti-cancer effect in melanomas since low CYP1B1 expression correlated with decreased invasive potential of endometrial cancer cells and increased sensitivity to specific anticancer drugs [43,44]. From the viewpoint of personalized medicine, these anti-tumor effects of CY1B1 downregulation can vary, since there are wide inter-individual differences in basal and induced levels of CYP1B1 in skin. Furthermore, CYP1A1 with similar functions as CYP1B1, may compensate for the downregulated CYP1B1 in both diseases [38].

**CYP3A5:** This enzyme metabolizes certain drugs and steroid hormones. Interestingly, we observed a 2–5 fold downregulation of CYP3A5 in psoriasis and melanoma samples relative to normal skin (Table 2 and Table 3). It is difficult to predict the impact of the downregulated CYP3A5 gene for two reasons. First, the substrate specificity of CYP3A5 is very similar to that of CYP3A4. Therefore, the CYP3A4 gene could compensate for decreased CYP3A5 expression in psoriasis. The second reason involves the different variants of CYP3A5. The Affymetrix probesets in the datasets we used recognize the wild type CYP3A5*1 allele which codes for the active CYP3A5 protein, and two variants. One of these variants is the CYP3A5*3/*3 allele which is common in Caucasians, and codes for a truncated, inactive protein [45,46]. If we assume that the samples of this study primarily came from Caucasian patients, then these probes have a high probability of binding the CYP3A5*3/*3 allele. Therefore, the observed downregulation of the CYP3A5 gene in psoriasis and melanoma samples may not have a significant impact on the drug metabolizing ability of keratinocytes and melanocytes in these two diseases.

### 3.11. Drug Development for Psoriasis and Melanomas

Although the levels and activities of CYP450 enzymes in skin are far lower than the levels of these enzymes in the liver [47], the CYP450 genes and enzymes in skin must be studied for two main reasons. First, the identification of expression patterns of CYP450 genes can help us understand how normal skin metabolizes drugs and detoxifies xenobiotics. Second, it is important to examine and compare the basal levels of CYP450 enzymes in diseased versus normal skin. Therefore, we analyzed four datasets on psoriasis and 3 datasets on melanomas to identify DEGs encoding CYP450 and phase II DMEs in these diseases relative to normal skin. The functional redundancy of certain CYP450 genes (CYP gene families1–4) makes it difficult to predict the effects of altered gene expression of the CYP1B1, CYP2J2, CYP3A4, and CYP3A5 DME genes identified by this study [1,2,47]. However, as explained below, some of our results have implications for new drug development strategies and conventional drug therapies used for these two diseases.

**Psoriasis:** Psoriasis is a TH-1 cell mediated, autoimmune disease involving over-expression of specific pro-inflammatory cytokines. Many DEGs in the psoriasis samples of this study are potential drug targets. For example, genes controlling abnormal keratinocyte differentiation and epidermal barrier function (CYP4F22, SULT2B1) were upregulated in psoriatic skin relative to normal skin (Figure 1, Table 2). Therefore, selective inhibition of the gene expression or enzyme activities of CYP4F22 and SULT2B1 can potentially normalize the stratum corneum, improve barrier function, and facilitate the penetration of drugs. Indeed, there are keratolytic drugs which improve the penetration of drugs into the psoriatic skin. However, drugs which specifically target the CYP4F22 and SULT2B1 genes may be more effective. Hyper-proliferation of skin cells is, in part, due to a deficiency of vitamin D. Therefore, vitamin D analogs which suppress proliferation of keratinocytes and Th1 and Th17 immune cells are widely used to suppress cell proliferation and inflammation in psoriasis [13]. We found that the gene controlling degradation of vitamin D (CYP24A1) was strongly upregulated in psoriasis samples (Figure 1, Table 2), and could be a novel drug target for psoriasis. Upregulation of CYP7B1, and the resulting increase in CYP7B1 enzyme activity can counteract the beneficial immune-suppressive effects of glucocorticoid therapy for psoriasis [29]. Therefore, selective inhibition of the gene expression or enzyme activity of CYP7B1, can potentially be used for personalized therapy of psoriasis patients undergoing glucocorticoid therapy. Although the CYP2C18 gene was upregulated in psoriasis, its potential can only be realized when clinically relevant dermatological drugs metabolized by the CYP2C18 enzyme are identified [35,36].

New anti-inflammatory drugs and biological therapies are being developed for psoriasis. Since suppression of CYP3A4 gene expression in inflammatory diseases can lead to improper drug metabolism and poor efficacy of therapeutic drugs, it is important to determine the effects of new therapies on CYP3A4 and other DME genes. Notably, biological therapies which target specific interferons (Pegasys; peginterferon α-2a) and the therapeutic monoclonal antibody targeting IL-6 receptor (tocilizumab), could de-repress the CYP3A34, CYP2C9, and CYP2C19 enzymes *in vitro* and in clinical studies [48,49]*.* Other biological therapies such as TNF-α antagonists did not suppress these CYP450 genes, but were reported to increase the risk of infections and other skin diseases [50].Therefore, conventional therapies (glucocorticoid therapy, vitamin D analogs) for psoriasis will continue to remain clinically relevant. Accordingly, the inhibition expression/activity of the CYP4F22, SULT2B1, CYP24A1, and CYP7B1 genes can potentially improve the efficacy of conventional therapies for psoriasis.

**Melanoma:** Three major DMEs (CYP1B1, CYP3A4, and CYP3A5) which metabolize a large percentage of anti-cancer drugs were downregulated in melanoma samples compared to normal skin. The impact of these downregulated genes on drug metabolism can vary, since these enzymes can be induced by specific drugs and xenobiotics. Upstream genes such as nuclear receptors, aryl hydrocarbon receptor, prostaglandins, and TNF-α can also induce these three genes [51]. Modern drugs for melanoma include signal transduction inhibitors which block the activity of proteins encoded by mutant B-Raf genes (Vemurafenib, dabrafenib, trametinib, and sorafenib) and angiogenesis inhibitors. Therefore, it is important to evaluate effects of these new drugs on activity of these three major DME enzymes (CYP1B1, CYP3A5, and CYP3A4). Notably, one phase I/II clinical trial found that patients given a specific dosage of sorafenib (400 mg twice daily for 28 consecutive days) did not show a significant change in CYP3A4 activity [52].

## 4. Conclusions

This is the first report to show that psoriasis and melanomas show opposite expression patterns of key CYP450 and phase II drug metabolizing genes when compared with normal skin. There are four novel findings from this data.

1. Genes controlling abnormal keratinocyte differentiation (CYP24A1) and epidermal barrier function (CYP4F22, SULT2B1) were up-regulated in psoriasis. Therefore, new drugs targeting these genes/enzymes could potentially improve drug penetration, and reduce inflammation and proliferation of keratinocytes and TH-1 cells in psoriatic skin.
2. Psoriasis samples showed upregulation of CYP7B1 which could result in increased breakdown of pro-inflammatory hydroxysterols. Melanomas showed the opposite trend. Thus, downregulation of CYP39A1 can lead to accumulation of pro-inflammatory hydroxysterols and enhance melanoma progression.
3. This relationship between CYP39A1 expression and inflammation is a novel link between two new hallmarks of cancer (abnormal lipid metabolism and inflammation).
4. Psoriasis samples have greater ability to metabolize drugs than melanoma samples. This is because the major DME (CYP3A4) was only down-regulated in melanoma samples.

## Figures and Tables

**Table 1:** Microarray datasets: Four datasets on psoriasis and three datasets on melanomas (GPL570 platform [HG-U133_Plus_2] Affymetrix Human Genome U133 plus 2.0 Array) were analyzed. Raw data was log-transformed, filtered, normalized, and annotated, to generate gene lists which were used for class comparison with BRB-Array tool. Abbreviations: NS (Normal Skin), PS (Psoriatic skin), PM (Primary Melanoma), DEG (Differentially expressed Genes).

Psoriasis	GSE 14905	GSE 30999	GSE 41662	GSE 34248
No. Arrays	21 (NS) 33 (PS)	85(NS) 85(PS)	24(NS) 24(PS)	14(NS) 14(PS)
No. Input Genes	4994	8961	12416	9596
No. DEG	3328	6826	4227	2186
**Melanoma**	**GSE15605**	**GDS1375**	**GSE46517**	
No. Arrays	16 (NS) 46 (PM)	7 (NS) 31 (PM)	7 (NS) 45 (PM)	
No. Input Genes	11938	6514	2378	
No. DEG	4279	3282	772	

**Table 2:** Differentially-expressed genes in plaque psoriatic skin *versus* normal skin. Mean fold change and *p*-values for each gene were calculated from three of the four datasets. The first seven genes were 3-25-fold upregulated in psoriasis. The last four genes were 1.80–2.50 fold downregulated in psoriasis.

Gene Symbol	Probe Set ID	Mean ± S.D Fold Change	Mean Parametric *p*-Value	Function of Enzyme Encoded by Gene	Impact of Altered Gene Expression On Disease
CYP27B1	205676_at	3.13 ± 0.43	3.20 × 10^−4^	Catalyzes activation of vitamin D	Vitamin D promotes normal keratinocyte differentiation
CYP24A1	206504_at	8.80 ± 0.68	9.00 × 10^−6^	Catabolizes active vitamin D	Depleted vitamin D leads to abnormal keratinocyte differentiation
CYP4F22	244692_at	5.00 ± 1.25	<1 × 10^−7^	Regulates hepoxillins in permeability barrier	Impaired corneocyte structure and barrier function
CYP7B1	207386_at	2.60 ± 0.80	9.02 × 10^−5^	Inactivates pro-inflammatory 25 and 27-hydroxysterols	Potential Anti-inflammatory effect. Interferes with action of Glucocorticoid Drugs
CYP2C18	208126_s_at	4.02 ± 0.89	2.32 × 10^−4^	Metabolizes important drugs and vitamin A	Vitamin A metabolites can inhibit keratinocyte proliferation
AKR1B10	206561_s_at	23.8 ± 15.80	<1 × 10^−7^	Trans-retinaldehyde-reductase degrades vitamin A	Decreased vitamin A can stimulate keratinocyte proliferation
SULT2B1	205759_s_at	3.31 ± 0.46	<1 × 10^−7^	Sulfo-transferase sulfates cholesterol	Excess cholesterol sulfate promotes abnormal barrier
CYP2J2	205073_at	0.37 ± 0.065	3.96 × 10^−4^	Produces EETs with anti-inflammatory effects.	Downregulation decreases EETs . Insufficient cornification and increased inflammation can occur
CYP4B1	210096_at	0.39 ± 0.04	1.26 × 10^−4^	Synthesizes eicosanoids. Activates specific xenobiotics	Downregulation has no impact since human CYP4B1 protein lacks function
CYP1B1	202436_s_at	0.37 ± 0.18	3.26 × 10^−4^	Activates specific xenobiotics and mutagens	Downregulation can protect against toxins and carcinogens
CYP3A5	205765_at	0.56 ± 0.05	9.73 × 10^−4^	Metabolizes important drugs	Downregulation can decrease drug metabolism

**Table 3:** Differentially Expressed Genes in Primary Melanomas *versus* Normal Skin. Mean Fold change and *p*-values for each gene were calculated from at least 2 of the 3 melanoma datasets. Five CYP450 genes and AKR1C3 and SULT2B1 genes were 3-11 fold downregulated in primary melanomas *versus* normal skin.

Gene Symbol	Probe Set ID	Mean ± SD Fold Change	Mean Parametric *p*-Value	Function of Enzyme Encoded by Gene	Impact of Altered Gene Expression On Disease
CYP4B1	210096_at	0.09 ± 0.05	<1 × 10^−7^	Synthesizes inflammatory eicosanoids. Activates specific xenobiotics	Downregulation has no impact since human CYP4B1 protein lacks function
CYP39A1	220432_s_at	0.27 ± 0.15	2.08 × 10^−4^	Inactivates pro-inflammatory 24-hydroxysterols	Downregulation can have pro-Inflammatory effect and increase metastasis
CYP1B1	202437_s_at	0.24 ± 0.11	1.56 × 10^−5^	Activates specific xenobiotics and mutagens	Downregulation can protect against toxins and carcinogens
CYP3A4	205999_x_at	0.31 ± 0.04	1.50 × 10^−5^	Metabolizes 50% of important drugs	Downregulation can significantly decrease drug metabolizing capacity
CYP3A5	214234_s_at	0.19 ± 0.15	3.33 × 10^−5^	Functionally similar to CYP3A4	Downregulation can decrease drug metabolism
AKR1C3	209160_at	0.19 ± 0.16	1.80 × 10^−5^	Trans-retinaldehyde-reductase which degrades vitamin A	Downregulation can stabilize vitamin A levels and limit cell proliferation
SULT2B1	205759_s_at	0.30 ± 0.14	5.95 × 10^−5^	Sulfo-transferase which sulfates cholesterol	Downregulation can lead to decreased proliferation
